# Correlation of Papillomacular Nerve Fiber Bundle Thickness with Central Visual Function in Open-Angle Glaucoma

**DOI:** 10.1155/2015/460918

**Published:** 2015-03-02

**Authors:** Wataru Kobayashi, Hiroshi Kunikata, Kazuko Omodaka, Kyousuke Togashi, Morin Ryu, Masahiro Akiba, Gaku Takeuchi, Tetsuya Yuasa, Toru Nakazawa

**Affiliations:** ^1^Department of Ophthalmology, Tohoku University Graduate School of Medicine, 1-1 Seiryo-machi, Aoba-ku, Sendai 980-8574, Japan; ^2^Department of Retinal Disease Control, Ophthalmology, Tohoku University Graduate School of Medicine, 1-1 Seiryo-machi, Aoba-ku, Sendai 980-8574, Japan; ^3^Department of Bio-Systems Engineering, Faculty of Engineering/Graduate School of Science and Engineering, Yamagata University, 4-3-16 Jonan, Yonezawa-shi, Yamagata 992-8510, Japan; ^4^Topcon Corporation, 75-1 Hasunuma-cho, Itabashi-ku, Tokyo 174-8580, Japan; ^5^Department of Advanced Ophthalmic Medicine, Tohoku University Graduate School of Medicine, 1-1 Seiryo-machi, Aoba-ku, Sendai 980-8574, Japan

## Abstract

*Purpose*. To determine the correlation of reduced retinal thickness in the central papillomacular bundle (CPB) to central visual function, including central retinal sensitivity and visual acuity, in glaucoma patients. *Methods*. This study enrolled 50 eyes of 50 patients with open-angle glaucoma who were carefully screened for comorbid conditions that can cause decreased central visual function, such as cataracts or macular diseases. We used a novel CPB analysis comprising a program for optical coherence tomography that measured RNFL thickness and GCC thickness in the CPB and divided lengthwise into three parts (upper, middle, and lower CPB). The relationship of these parameters, including conventional macular thickness, to visual field sensitivity in four central standard automated perimetry points (the central four thresholds) and BCVA was analyzed. *Results*. The two parameters most highly correlated with central four thresholds were macular GCCT and macular RNFLT. The two parameters most highly correlated with BCVA were middle CPB (mid-CPB) GCCT and mid-CPB RNFLT. A multiple regression analysis revealed that mid-CPB GCCT was an independent factor impacting central retinal thresholds and BCVA. *Conclusions*. Our results suggest that mid-CPB RNFLT and GCCT, parameters of a novel papillomacular bundle analysis, are candidate biomarkers of decreased central visual function in glaucomatous eyes.

## 1. Introduction

Glaucoma affects over 70 million people worldwide and is currently the second most common cause of blindness [1, 2]. It is characterized by reduction of the retinal ganglion cell layer (GCL) and loss of the axons comprising the retinal nerve fiber layer (RNFL) [3]. The incidence of glaucoma increases with age [4], and glaucoma-induced visual disorder is becoming an increasingly serious problem in an aging society. A major risk factor for glaucoma is high intraocular pressure (IOP), and treatment to lower IOP is commonly recommended to patients with glaucoma [5, 6].

Macular functions, including visual acuity (VA) and retinal sensitivity, are very important for quality of life even in patients with glaucoma [7]. Though VA is generally preserved until the late stages of glaucoma [8], cases have also been reported of patients whose VA decreased even in the early stages of the disease [9, 10]. Recently, spectral domain optical coherence tomography (SD-OCT) technology has been introduced, which enables us to visualize and quantify each retinal layer in the macular area, returning parameters such as ganglion cell complex thickness (GCCT), with programmed segmentation algorithms [11]. Several studies have reported that the diagnostic power for glaucoma of measurements of macular GCCT and circumpapillary retinal nerve fiber layer thickness (cpRNFLT) is very similar [12, 13]. Furthermore, our previous research revealed that mid-temporal cpRNFLT is significantly correlated to VA in patients with glaucoma (*r* = −0.40) and the predictive accuracy for the presence of decreased VA was also high (the area under the curve (AUC) for the receiver operating characteristic (ROC) was 0.79) [14].

We hypothesized that there may be thinning of the central papillomacular bundle (CPB) in glaucomatous eyes with decreased central visual function. To evaluate this hypothesis, we first measured CPB thickness with a newly developed OCT analysis program and then evaluated the correlation between the CPB and macular thickness (including RNFLT and GCCT) and central visual function including VA and central retinal sensitivity measurements made with standard automated perimetry (SAP). Thus, the purpose of this study was to evaluate the relationship between the CPB and macular thickness and VA and central retinal sensitivity in eyes with glaucoma.

## 2. Subjects and Methods

### 2.1. Inclusion Criteria

This retrospective, cross-sectional study comprised 50 eyes of 50 Japanese adult patients with open angle glaucoma (OAG). All the patients exhibited glaucomatous optic neuropathy. The inclusion criteria were (1) diagnosis of OAG, including primary open angle glaucoma (POAG) and normal tension glaucoma (NTG); (2) age > 40 years old; (3) a spherical equivalent refractive error of >−8.00 diopters; (4) a glaucomatous visual field meeting the Anderson-Patella classification [15]. The exclusion criteria were (1) decimal visual acuity <0.1, (2) cataracts with severity greater than grade 2 of the Emery-Little classification, and (3) macular disease such as macular edema, macular degeneration, or epiretinal membrane. The baseline clinical parameters recorded for each patient were age, sex, and refractive error. The baseline best-corrected VA (BCVA) was measured with a standard Japanese decimal visual acuity chart and converted to logarithm of the minimum angle of resolution (logMAR) for statistical analysis. IOP was measured by Goldmann applanation tonometry at the time of the initial diagnosis of OAG before the use of any medications for glaucoma. The study adhered to the tenets of the Declaration of Helsinki, and the protocols were approved by the Clinical Research Ethics Committee of Tohoku University Graduate School of Medicine.

### 2.2. Visual Field Analysis

Mean deviation (MD) values were obtained with the Swedish interactive threshold algorithm- (SITA-) standard strategy of the 30-2 program of the Humphrey field analyzer (HFA; Carl Zeiss Meditec, Dublin, CA, USA). HFA examinations were performed within three months of the OCT measurements. Only reliable MD values were used, defined as those with <20% fixation errors and <33% false-positives or -negatives. Central retinal sensitivity was defined as mean visual field sensitivity in the four central standard automated perimetry points (the central four thresholds).

### 2.3. OCT Macular Area

We measured macular RNFLT and GCCT with 3D OCT-2000 software (version 8.00; Topcon Corporation, Tokyo, Japan). After obtaining macular cube scans (7 × 7 square mm, corresponding to a 10-degree square area of the retina in the macula) centered on the fovea, the embedded 3D OCT program was used to calculate the thickness of each layer automatically.

### 2.4. OCT Central Papillomacular Bundle

**Figure 1:**
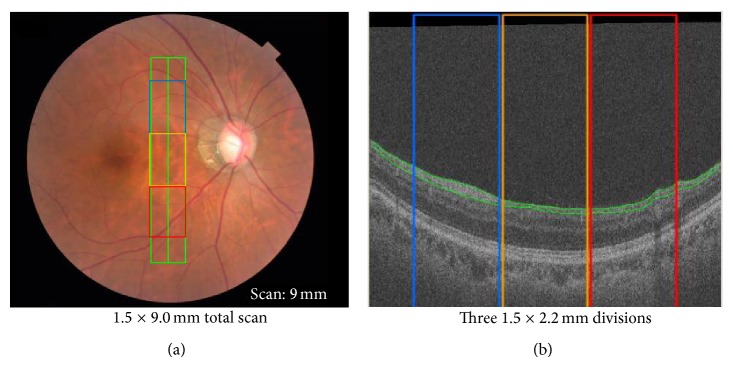
Fundus and optical coherence tomography images of the central papillomacular bundle: a rectangular area centered between the optic nerve disc and the macula, with three lengthwise divisions. (a) Fundus image showing the rectangular scanning area, which is centered between the optic nerve disc and the macula and aligned perpendicularly to the nerve fibers. (b) Representative OCT B-scan image showing sagittal segmentation results. The RNFLT and GCCT were measured in 3 equally sized 1.5 × 2.2 mm sections of the scanning area.

The CPB was defined in this study as a 1.5 × 9.0 mm rectangular area centered between the optic nerve disc and the macula, aligned perpendicularly to the nerve fibers (Figure 1(a)). At either end of the scan area, a 1.5 × 1.2 mm area in which the retinal layers could not be reliably segmented was discarded. The remaining 1.5 × 6.6 mm area was divided lengthwise into three 1.5 × 2.2 mm sections, representing the upper, middle, and lower CPB (Figure 1(b)). The analysis used 3D OCT images, obtained with the 3D OCT-2000 (Topcon corp.) device. Each image was constructed from 64 B-scan images (pixel dimensions: 512 × 885, grayscale levels: 256) with depth and lateral resolutions of 6 *μ*m and 20 *μ*m, respectively. Layer segmentation was performed with newly developed software (Topcon corp.). The RNFLT and GCCT of the CPB were measured with automatic analysis software developed by the Graduate School of Science and Engineering, Yamagata University. This software was equipped with a registration system (using a fast registration algorithm for the 3D OCT images based on en face projection images) to ensure the reliability and reproducibility of the clinical data.

### 2.5. Statistical Analysis

Spearman's correlation analysis was used to determine the correlation between data from the structural examination, including RNFLT and GCCT, and data from the functional examination, including BCVA, MD, and the central four thresholds. A multiple regression analysis was used to determine the correlation of the central four thresholds and BCVA to age, IOP, refractive error, and mid-CPB GCCT. Our statistical analysis relied on the JMP Pro version 9.0.2 software for Windows (SAS Institute, Japan). A *P* value <0.05 was considered to be statistically significant.

## 3. Results

**Figure 2:**
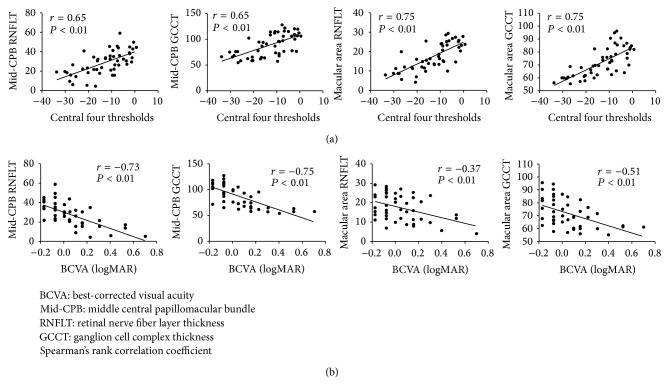
Correlation of the central four thresholds and best-corrected visual acuity (BCVA) to retinal nerve fiber layer thickness and ganglion cell complex thickness in the middle central papillomacular bundle (mid-CPB) and macular area. Scatterplots of the correlation of the central four thresholds to the mid-CPB and macular thickness (a) and scatterplots of the correlation of BCVA to the mid-CPB and macular thickness (b).

We set out to study the correlation of our new CPB analysis with decreased central visual function in glaucoma patients. Characteristics and clinical findings of the 50 eyes with glaucoma enrolled in this study are shown in Table 1. The correlation of retinal thickness with VA and retinal sensitivity is shown in Table 2. Strong correlations of more than *r* = 0.7 were detected between BCVA and both RNFLT and GCCT in the middle CPB (mid-CPB) (*r* = −0.73, *P* < 0.01 and *r* = −0.75, *P* < 0.01, resp.) and between the central four thresholds and both RNFLT and GCCT in the macular area (*r* = −0.75, *P* < 0.01 and *r* = −0.75, *P* < 0.01, resp.). Mid-CPB parameters were more closely correlated with VA and the four central thresholds than with MD. A multiple regression analysis revealed that macular GCCT was an independent factor impacting the central four thresholds and BCVA (*P* < 0.01, Table 3, and *P* < 0.01, Table 4, resp.).  Figure 2 shows scatterplots for the correlation of the central four thresholds and BCVA to the mid-CPB and macular thickness.

## 4. Discussion

We set out to determine the ability of our new CPB analysis to predict decreased central visual function in glaucoma patients. Among the parameters comprising the new analysis, we found that mid-CPB GCCT had the strongest correlation with BCVA, as well as a strong correlation with the central four thresholds. A multiple regression analysis further revealed that mid-CPB GCCT was an independent factor impacting BCVA and the central four thresholds. Among the new indicators in our analysis, therefore, mid-CPB GCCT has great potential to detect decreased central visual function in eyes with glaucoma.

There have not yet been any studies of the relationship between the thickness of each layer of the macula and visual acuity, although a previous study has reported that mean total macular thickness was significantly decreased in patients with poor BCVA [16]. Our study now confirms that both macular RNFLT and GCCT are also correlated with BCVA (*r* value: approximately −0.4~0.5), although being weakly correlated. In addition, interestingly, we found that RNFLT and GCCT in the mid-CPB were both strongly correlated with BCVA (*r* value: approximately 0.7). We believe that these results are consistent with the current understanding of glaucoma, because progression of the disease in the ganglion cell complex involves thinning of the papillomacular bundle and macular lesions that are detectible with OCT and can manifest as decreased VA. It is unclear why the correlation of BCVA and retinal thickness is better in specific CPB layers than in the macula, but it may be that, generally, VA-related structures, including the RNFL and the GCL, are more concentrated in the mid-CPB than in the macula. This indicates a close relationship between the structure of the mid-CPB and VA in eyes with glaucoma.

Our study also supports existing data showing that indices of retinal sensitivity such as MD are correlated with RNFLT and GCCT in the macular area [12, 17–20]. We found that GCCT and RNFLT in the macular area were strongly correlated with central four thresholds sensitivity (*r* value: approximately 0.7~0.8), and that GCCT and RNFLT in the mid-CPB were moderately correlated with central four thresholds sensitivity (*r* value: approximately 0.6). It is unclear why central sensitivity is better correlated to the thickness of these specific macular layers than to CPB thickness, but it may be that, generally, the lower temporal cpRNFLT is especially susceptible to thinning in some cases of glaucoma. We speculate that sensitivity in the lower temporal point of the central four thresholds decreases with the reduction of cpRNFLT and that this is a critical influence on the central four thresholds in these cases.

The advantage of the new CPB area in our analysis is that it contains a large portion of the papillomacular nerve fiber bundle that extends to the macula, especially the fovea. It is well known that, despite the effect of glaucoma on VA in the final stages of the disease, it does not usually affect the macula in the early stages [8]. However, earlier reports have also shown that in some cases the macula and CPB may be affected even in the early stages of glaucoma [9, 10, 21, 22]. Generally, VA depends on foveal function, and though each layer of the retina in the foveal region contains a different section of the ganglion cells, the lack of a GCC in the fovea is an adaptation allowing for higher VA. The RNFL contains the axons of the cells, the combined ganglion cell and inner plexiform layers (GCL + IPL) contain the cell bodies and dendrites, and the GCC contains all these sections (RNFL + GCL + IPL). Degeneration of the axons and dendrites has been detected in glaucoma patients [23]. After this degeneration, microglia phagocytose the resulting debris and the thickness of the RNFL immediately decreases before subsequent GCL loss. This suggests that functional deterioration may be better correlated with axonal loss than with cell body loss of the retinal ganglion cell in the macular area. Thus, a high rate of retinal ganglion cell death, approximately 20%, is necessary before at least a 5 dB decrease in SAP measurements of MD can be detected [3]. Furthermore, it has also been demonstrated that the RNFL [24], GCC [12], and GCL + IPL [25] are thin or absent in the fovea and do not have clearly demarcated borders. This makes it very difficult to perform automatic segmentation of the retinal layers near the macula and leads to errors. We believe that this also explains the strength of the correlation of the GCCT and RNFLT in the mid-CPB to BCVA, that is, foveal function.

Our study had several limitations. It included only a small number of glaucomatous eyes. Additionally, biases including age, axial length, stage of glaucoma, and myopia have been reported to affect determinations of the correlation between structure and function. To minimize bias, we excluded high myopic patients with glaucoma (less than −8 diopter) and confirmed, with a single regression analysis, that neither the strength of myopia nor axial length was associated with any parameters of the macular area.

In conclusion, we found that mid-CPB structure, the central part of a novel papillomacular bundle analysis, was strongly correlated with BCVA and central sensitivity. These results suggest that this new CPB analysis may be useful as a noninvasive objective evaluation capable of identifying decreased central visual function in glaucoma patients.

## Figures and Tables

**Table 1:** Characteristics of patients.

Characteristics	Number, mean ± SD
Glaucoma type	
POAG	22
NTG	28
Sex	
Male	28 (28 eyes)
Female	22 (22 eyes)
Laterality	
Right	19
Left	31
Age (years)	61.8 ± 9.4
BCVA (logMAR)	0.04 ± 0.2
IOP (mmHg)	14.4 ± 3.4
Refractive error (diopter)	−3.28 ± 2.6
HFA 30-2 mean deviation (MD; dB)	−12.3 ± 8.0
Central four thresholds (dB)	−13.1 ± 9.1
Macula area	
RNFLT (*μ*m)	17.4 ± 6.9
GCCT (*μ*m)	72.1 ± 11.0
Central papillomacular bundle	
RNFLT (*μ*m)	
Upper	30.3 ± 18.0
Middle	29.0 ± 12.2
Lower	30.5 ± 17.1
GCCT (*μ*m)	
Upper	76.3 ± 20.2
Middle	88.5 ± 21.7
Lower	75.2 ± 18.0

POAG: primary open angle glaucoma, NTG: normal tension glaucoma, BCVA: best-corrected visual acuity, logMAR: logarithm of the minimal angle resolution, IOP: intraocular pressure, HFA: Humphrey field analyzer, RNFLT: retinal nerve fiber layer thickness, and GCCT: ganglion cell complex thickness.

**Table 2:** Correlation of retinal thickness with visual acuity and retinal sensitivity.

	Upper CPB		Mid-CPB		Lower CPB		Macular area	
	RNFLT	GCCT	RNFLT	GCCT	RNFLT	GCCT	RNFLT	GCCT
BCVA	−0.26	−0.25	−0.73^**^	−0.75^**^	−0.24	−0.28^*^	−0.37^**^	−0.51^**^
Total MD	0.48^**^	0.52^**^	0.42^**^	0.46^**^	0.44^**^	0.40^**^	0.66^**^	0.62^**^
Central four thresholds	0.62^**^	0.57^**^	0.65^**^	0.65^**^	0.59^**^	0.59^**^	0.75^**^	0.75^**^

CPB: central papillomacular bundle, RNFLT: retinal nerve fiber layer thickness, GCCT: ganglion cell complex thickness, BCVA: best-corrected visual acuity, and MD: mean deviation.

Spearman's rank correlation coefficient, ^*^ *P* < 0.05, ^**^ *P* < 0.01.

**Table 3:** Results of multiple regression analysis for factors independently impacting the central four thresholds.

Variables	Partial regression coefficient	Standardized partial regression coefficient	*P* value
Age	−0.0500	−0.0514	0.7127
IOP	0.0977	0.0360	0.7660
Ref	−0.7759	−0.2213	0.0916
Mid-CPB GCCT	0.2568	0.6110	<0.01

IOP: intraocular pressure, Ref: refractive error, Mid-CPB: middle central papillomacular bundle, and GCCT: ganglion cell complex thickness.

Multiple correlation coefficient: *r* = 0.6894; coefficient of determination: *r* ^2^ = 0.4753.

**Table 4:** Results of multiple regression analysis for factors independently impacting best-corrected visual acuity.

Variables	Partial regression coefficient	Standardized partial regression coefficient	*P* value
Age	0.0017	0.0809	0.5472
IOP	−0.0072	−0.1214	0.2994
Ref	−0.0105	−0.1372	0.2723
Mid-CPB GCCT	−0.0062	−0.6717	<0.01

IOP: intraocular pressure, Ref: refractive error, Mid-CPB: middle central papillomacular bundle, and GCCT: ganglion cell complex thickness.

Multiple correlation coefficient: *r* = 0.7174; coefficient of determination: *r* ^2^ = 0.5146.
